# Ceramide/protein phosphatase 2A axis is engaged in gap junction impairment elicited by PCB153 in liver stem-like progenitor cells

**DOI:** 10.1007/s11010-021-04135-z

**Published:** 2021-04-10

**Authors:** Roberta Squecco, Federica Pierucci, Eglantina Idrizaj, Alessia Frati, Elena Lenci, Catia Vicenti, Maria Chiara Iachini, Maria Martinesi, Rachele Garella, Maria Caterina Baccari, Fabio Francini, Elisabetta Meacci

**Affiliations:** 1 Department of Experimental and Clinical Medicine, Section of Physiological Sciences, University of Florence, Viale GB Morgagni 63, 50134 Florence, Italy; 2 Department of Experimental and Clinical Biomedical Sciences “Mario Serio”, Research unit of Molecular and Applied Biology, University of Florence, Viale GB Morgagni 50, 50134 Florence, Italy

**Keywords:** Environmental pollutant PCB153, Connexin, Ceramide, Gap junctions, Protein Phosphatase 2A, Sphingolipids

## Abstract

The widespread environmental pollutant 2,2′,4,4′,5,5′-hexachlorobiphenyl (PCB153) is a non-dioxin-like toxicant. It is a potential carcinogen compound able to induce gap junction (GJ) intercellular communication impairment, probably the first non-genomic event leading to tumor promotion. Although PCBs have been known for many years, the molecular mode of PCB153 action is still unclear. Recent studies from our research group have shown that the toxicant elicits a transient modulation of connexin (Cx) 43-formed GJs in hepatic stem-like WB-F344 cells involving sphingosine 1-phosphate (S1P) path. Taking into account that other strictly related bioactive sphingolipids, such as ceramide (Cer), may have different effects from S1P, here we aim to clarify the signaling paths engaged by PCB153 in the control of GJs, focusing primarily on the role of Cer. Accordingly, we have achieved a combined biomolecular and electrophysiological analysis of GJs in cultured WB-F344 cells treated with PCB153 at different time points. We have found that the toxicant elicited a time-dependent regulation of GJs formed by different Cx isoforms, through a transient modulation of Cer/Cer kinase (CerK) axis and, in turn, of protein phosphatase 2A (PP2A). Our new findings demonstrate the existence of a specific molecular mechanism downstream to Cer, which distinctly affects the voltage-dependent and -independent GJs in liver stem-like cells, and open new opportunities for the identification of additional potential targets of these environmental toxicants.

**Supplementary Information:**

The online version contains supplementary material available at 10.1007/s11010-021-04135-z.

## Introduction

Among environmental pollutants, polychlorinated biphenyls (PCBs) represent a serious problem for human health. Large amounts of PCB compounds have been produced in many countries for industrial applications over several decades. PCBs are extremely inert chemicals with properties that make them useful in several commercial and industrial applications such as heat transfer, electrical and hydraulic equipment, in plastics [1]. PCBs production has been banned since 1977 because of their adverse effects on the health of humans and animals. Although no known industrial consumer product currently contains PCBs, manufacturing still releases them. PCBs accumulate and persist in the environment due to chemical or biological processes (i.e., volatilization, partitioning). Moreover, the higher chlorinated PCB congeners percolate into the soil, where they tend to remain with half-lives of months or years. Nowadays, the general population may be exposed to PCBs from a variety of sources, such as diet and the environment. However, the main route of exposure for humans has been, and continues to be, the ingestion of contaminated food [2]. In fact, PCBs can accumulate in the food chain [3]. Other sources of toxicant exposure are inhalation and dermal routes, occupational exposures in buildings containing PCBs, or during PCB clean-up activities. Bioaccumulated PCBs persist in the body. Since highly chlorinated congeners are more slowly metabolized, they accumulate to higher levels especially in fatty tissues, and therefore, they are of special relevance to human health. In fact, they have a variety of adverse effects, such as endocrine disruption, developmental neurotoxicity, and carcinogenicity [4, 5]. Most PCB exposures are a mixture of non-coplanar and coplanar toxicants. Although coplanar PCBs seem to play a key role in tumor-promoting effects of PCB mixtures (i.e., Aroclor 1260) through the activation of aryl hydrocarbon receptor (AhR), the non-coplanar fraction of this blend also contributes considerably to its tumor-promotion potential [6]. Understanding the underlying mechanisms of the non-genotoxic tumorigenic potential of the non-coplanar PCBs is essential for a thorough evaluation of the risk that environmentally relevant PCBs represent for human health.

An important role in protecting the organism from possible injury due to chemical toxicants is played by the liver and its ability to change lipophilic substances into more water-soluble metabolites, which can be efficiently removed from the body through the urine [7]. Liver, as the lead site for xenobiotic metabolism, is a crucial target of PCBs harmful action [8]. In this regard, hepatic tissue and/or liver cell lines are of special relevance when analyzing the effects of PCBs.

Many tumor-promoting agents act by down-regulating the gap junctional intercellular communication (GJIC). Hampering the delivery of growth suppression signals sent from the neighboring cells [9, 10] leads to the progressive isolation of the initiated cell. Consequently, the inhibition of GJIC can be supposed to be a typical marker of tumor-promoting strength for a given compound. In this regard, previous in vitro studies investigated the relative potencies of different PCB congeners to inhibit GJIC in the rat liver epithelial oval cell line WB-F344 [11]. Among the PCB compounds exerting detrimental actions in the environment, usually the non-coplanar toxicants seem to be the strongest inhibitors of GJIC. Noteworthy, a structure-dependent activity has been extensively reported for different PCB congeners especially for dioxin-like (DL) PCBs, such as PCB126 (3,3′,4,4′,5-Pentachlorobiphenyl, planar congener), compared to the non-dioxin-like (NDL) PCBs153 (2,2′,4,4′,5,5′-hexachlorobiphenyl, di-ortho-substituted nonplanar congener) in different preparations and on different biological effects [12].

Nowadays, the molecular mechanisms involved in the NDL-compound PCB153 toxic action have not been fully clarified. PCBs affect the cell membrane organization and functions [13] and, in particular, PCB153 induces a rapid and sustained inhibition of GJIC and some other relevant alterations in rat liver epithelial stem-like cell line WB-F344 [11, 14, 15].

The gap junctions (GJs) are channels connecting two adjacent cells able to mediate the intercellular exchanges of different molecules, such as second messengers, inorganic ions, and other regulatory substances involved in a variety of cellular events, like cell proliferation and differentiation and/or pathological events, such as carcinogenesis [16, 17]. GJs are made of two docking hemichannels (or connexons), consisting of proteins named connexins (Cxs) that may have different molecular weights [18]. When the Cx isoforms are of the same type within a hemichannel, the resulting structure is called homomeric, whereas it is called heteromeric if more than one Cx isoform is present. The GJs made of two identical hemichannels are named homotypic, and those consisting of two different hemichannels are named heterotypic. The gating properties of these types of GJs are not the same and depend on the composition of Cx isoforms and on their voltage sensitivity [19]. In fact, some Cx isoforms (i.e., Cx43, Cx32, and so on) are more voltage dependent than others that, in contrast, are scarcely voltage dependent (i.e., Cx26). The latter isoform, also known as GJB2, is voltage dependent only at voltages greater than the physiological one (about ± 100 mV) [20].

Along with determining the voltage dependence of the GJ channel opening, the type of Cxs is also responsible for the protein regulation and the GJ gating modulation through other mechanisms. Indeed, Cxs can be regulated via phosphorylation by various protein kinases, such as PKC [21, 22]. Interestingly, Cx phosphorylation is a key mechanism of GJ regulation and is implicated in several stages of the Cx “lifecycle,” such as the trafficking, assembly/disassembly, degradation as well as the gating of GJ channels [18, 21, 23].

The GJIC can be modulated by bioactive sphingolipids (SLs), bioactive molecules involved in regulating a wide spectrum of biological processes, such as apoptosis, differentiation, and proliferation but also in oncogenesis, cancer progression, and inflammation [24, 25]. Particularly, sphingosine (Sph) and ceramide (Cer), and their related phosphorylated forms, sphingosine 1-phosphate (S1P), and ceramide 1-phosphate (C1P) can be important determinants for cell fate and for carcinogenesis [26]. In particular, sphingosine kinase (SphK) and ceramide kinase (CerK) promote the phosphorylation of sphingosine to S1P and ceramide to C1P. It is worth mentioning that pushing the balance toward the phosphorylated form represents a cell survival signal and a proliferative cellular response. Indeed, SphK is an oncogene [27–29]. On the other hand, one of the negative regulators of SphK activity, the Ser/Thr phosphoprotein phosphatase PP2A, is a tumor suppressor enzyme implicated in cell transformation of various cancers, including hepatocellular carcinoma [26] as well as many other cellular events [30–32]. Some studies report that PP2A activity is positively regulated by Cer [33, 34]. In addition, the involvement of bioactive SLs in the regulation of the GJs has been reported in various in vitro and in vivo models [35–37]. In this regard, recent findings from our research group have indicated that sphingolipid metabolism and, in particular, SphK/S1P axis are involved in PCB153 effects on GJ in WB-F344 cells [15]. Based on these premises, the present study aims to further elucidate the mechanisms of action of PCB153 on the GJ functionality in liver epithelial stem-like cell line WB-F344. Particularly, we intend to focus on the role of ceramide and PP2A in the modulation of the voltage-dependent and -independent GJs. Among the voltage-dependent Cx isoforms, we will mainly consider Cx43 expression, which is highly regulated in the pathological conditions induced by a variety of chemical and biological toxicants able to suppress hepatocellular GJIC [16].

## Materials and methods

### Cell culture and chemical treatment

Rat liver epithelial stem-like WB-F344 cells, non-tumorigenic epithelial cell line, kindly provided by Dr. JE. Trosko (Michigan State University, East Lansing, MI, USA) [13], were cultured in plastic dishes (Sigma Aldrich, Milan, Italy) as reported in [14, 15]. Cells were maintained in Dulbecco’s modified Eagle’s Medium (DMEM) (Sigma, Milan, Italy) supplemented with 1% L-glutamine, 1% penicillin/streptomycin (Sigma, Milan, Italy), and 10% fetal bovine serum (FBS) (Sigma, Milan, Italy). The specific inhibitor of PP2A, Cantharidin (Cant 3 μM), and the ceramide kinase (CerK) non-competitive inhibitor K1 (iCK, 10 μM) (Tocris, Bristol, UK) [38–40] were dissolved in 100% DMSO (Sigma Aldrich, Milan, Italy). All the inhibitors were added to the cell medium prior PCB153 exposure. PCB153 (Sigma Aldrich, Milan, Italy) was dissolved in DMSO and used at final concentration of 20 µM, an efficient overthreshold dose and a non-cytotoxic concentration as already reported [15]: significant changes in GJIC level were reached at the concentration of 20 μM and, to a lesser extent, at 10 μM, but not at 5 μM PCB153. No effects were observed in GJIC in cells treated with 5 μM PCB153. The final concentration of DMSO (vehicle) in all experiments was less than 0.1% (v/v)**.** Cells were treated with PCB153 for the indicated time. Cell permeable synthetic analog of ceramide, D-*erythro*-C8-ceramide (8 μM), was dissolved in 100% DMSO and was from Sigma Aldrich (Milan, Italy). Subconfluent cells were treated with all these compounds for time intervals indicated in the figures.

### Electrophysiological records

The functionality of the GJIC between WB-F344 cell pairs was tested by using the dual whole-cell patch clamp as previously described [15, 36, 41–43]. Coverslips with adherent cells in the recording chamber were superfused with a normal Tyrode bath solution containing (mM) 140 NaCl, 5.4 KCl, 1.8 CaCl_2_, 1.2 MgCl_2_, 10 D-glucose, and 5 HEPES (pH 7.4 with NaOH). The patch pipettes were filled with a filling solution containing (mM) 150 CsBr, 5 MgCl_2_, 10 EGTA, and 10 HEPES (pH 7.2 with tetraethylammonium-OH). All the chemicals were purchased from Sigma Aldrich, Italy. The patch pipettes were pulled from borosilicate glass (GC 150-15; Clark, Reading, UK). Pipettes resistance was 1.3–1.7 MΩ. Experiments were made at 22 °C. The electronic set up used consisted of an Axopatch 200B amplifier (Axon Instruments, Union City, CA), an analog-to-digital/digital-to-analog interface (Digidata 1200; Axon Instruments, Union City, CA), and pClamp 6 software (Axon Instruments, Union City, CA). Currents were low pass filtered at 1 kHz with a Bessel filter; the sampling interval was 0.6 ms.

The protocol of the stimulation in voltage clamp and the recording procedure have been previously reported [41] and detailed in the supplemental files. In brief, from a holding potential (HP) of 0 mV*,* cell *1* of the pair was stepped using a bipolar 5-s pulse protocol starting at transjunctional voltage *V*_j_ = ± 10 mV and ongoing at 20-mV increments up to ± 150 mV. The transjunctional current is indicated as *I*_j_. Precisely, the amplitude of *I*_j_ determined at the end of each pulse is indicated as steady-state current, *I*_j,ss_. These values were used to calculate the related conductances *G*_j,ss_. The *G*_j,ss_ voltage dependence was represented by the *G*_j,ss_/*V*_j_ plot that was best fitted by the Boltzmann function.

### RT-PCR

*RNA isolation*—Total RNA was isolated by extraction with TRIREAGENT (Sigma Aldrich, Milan, Italy) according to the manufacturer’s protocol and as reported in [44, 45]. Ratio of readings at 260 nm and 280 nm (260/280) provides an estimate of purity of RNA and was measured using a Nanodrop (Thermo Scientific, Waltham, Massachusetts, USA). Pure RNA had the absorbance ratio 260/280 between 1.8 and 2.0.

*Reverse transcription and PCR analysis*—1 μg of total RNA from WB-F344 cells was reverse transcribed to cDNA using SuperScript® III cells Direct cDNA Synthesis Kit (Life Technologies, Europe BV, Monza Italy) according to the manufacturer’s instructions. The reaction conditions were incubated for 10 min at 25 °C, 120 min at 37 °C, 5 min at 85 °C and then the samples were stored at − 20 °C. Quantitative real-time PCR was carried out using 7500 Fast Real-Time PCR System (Applied Biosystems, CA, USA) and/or in MIC Diatech Pharmacogenetics (Ancona, Italy) according to these thermal conditions: 95 °C for 10 min, followed by 40 cycles at 95 °C for 30 s, 52–60 °C for 30 s, 72 °C for 45 s (fluorescence was collected during the elongation step), finally followed by 95 °C for 15 s, 60 °C for 60 s, 95 °C for 15 s, and 60 °C for 15 s for the dissociation analysis.

The PCR reaction was performed in 25 μl volume and consisted of cDNA 100 ng, 0.2 μM of each primers and 12.5 μl of Power SYBR Green PCR Master Mix (Life technologies, Europe BV, Monza Italy) as reported in the table [44, 45]. Both positive and negative controls were included in each assay.

In order to amplify the Cx isoforms and GAPDH housekeeping genes, we used specific forward and reverse primers (Primers stocks 100 μM, Sigma Aldrich, Milan, Italy) reported below:

**Table:** 

Genes	Sequence (5′-3′)	Annealing temperature (°C)
GAPDH forward	GGCAAATTCAACGGCACAGTC	52
GAPDH reverse	TCGCTCCTGGAAGATGGTG	52
Cx43 forward	AACAGTCTGCCTTTCGCTGT	59
Cx43 reverse	TCTGCTTCAGGTGCATCTCC	59
Cx32 forward	AAAATGCTACGGCTTG AGGG	60
Cx32 reverse	TGAAGACGGTTTTCTCG GTG	60
Cx26 forward	ACTCCACCAGCATTGG AAAG	60
Cx26 reverse	TGAGAGATG GGGAAGTGGTG	60

The results of the real-time PCR were presented as ΔCt values where ∆Ct is the difference in Ct between the target gene and the housekeeping one. All values were normalized to the GAPDH housekeeping gene expression.

### Western blotting

Western blotting was performed to determine protein levels of Cx43, Cx32, and Cx26 in WB-F344 cells. Following the indicated treatments, cells were lysed at 4 °C with RIPA buffer containing 50 mM TrisHCl, pH 7.5, 120 mM NaCl, 1 mM EDTA, 6 mM EGTA, 15 mM Na_4_P_2_O_7_, 20 mM NaF, 2 mM Na_3_VO_4_, 1% Nonidet, and protease inhibitor cocktail (1.04 mM AEBSF, 0.08 mM aprotinin, 0.02 mM leupeptin, 0.04 mM bestatin, 15 mM pepstatin A, 14 mM E-64) (Sigma Aldrich, Milan, Italy). Lysates were centrifuged at 500 × *g* for 5 min at 4 °C, and protein concentration was measured using the Bradford microassay (Bio-Rad, Hercules, CA). Aliquots containing 10–15 μg of proteins were diluted in 2 × loading buffer (10% SDS, 50% glycerol, 0.25% bromophenol blue, and 0.25 M Tris–HCl, pH 6.8) and reduced at 90 °C for 10 min. Samples were subjected to 12–14% SDS–polyacrylamide gel electrophoresis (SDS-PAGE) and transferred onto a nitrocellulose membrane (Hybond ECL membranes, GE Healthcare; Little Chalfont, UK) as previously reported [15, 39].

The blots, after sufficient washes with phosphate buffer saline PBS, were first blocked with PBST buffer (150 mM NaCl, 0.05% Tween-20, and 20 mM Tris–HCl, pH 7.4) containing 5% non-fat dry milk (Bio-Rad, Hercules, CA) for 1 h at room temperature. After blocking, the membranes were washed in PBST and then incubated overnight with a 1:1000 dilution of following primary antibodies:

rabbit polyclonal anti-Cx43, mouse monoclonal anti-PP2A clone 1D6 (Upstate Biotechnology, Inc., NY USA), mouse monoclonal β-tubulin and anti-Cx26 (Sigma Aldrich, Milan, Italy), mouse monoclonal anti-Cx32, and anti-β-actin antibodies (Santa Cruz Biotechnology, Dallas, Texas, USA). After sufficient washes with PBST, the blots were incubated for 1 h at room temperature with goat anti-rabbit or goat anti-mouse horseradish peroxidase-conjugated secondary antibody (Santa Cruz, Texas, USA) at a 1:10.000/1:5.000 dilution.

Following three washes with PBST, immunoreactive protein bands were visualized using ECL Western Blotting Detection Reagents (GE Healthcare, Buckinghamshire, UK) or Lumina from Millipore (Billerica, MA, USA) and finally, the blots were exposed to high-performance chemiluminescence film (GE Healthcare, Buckinghamshire, UK). The identity of the bands on the western blots was confirmed by comparing to the precision marker molecular weight marker (Bio-Rad, Hercules, CA).

### PP2A enzymatic activity determination

PP2A enzymatic activity was determined by PP2Ac immunoprecipitation and phosphatase assay based on the immunoprecipitation of the enzyme using anti-PP2Ac Ab (clone 1D6; Upstate Biotechnology, Inc., NY USA) and malachite green reaction according to Immunoprecipitation Phosphatase Assay Kit protocol (Upstate Biotechnology, Inc., NY USA) with minor modifications, as previously reported [15]. Released phosphate was quantified by reading at 650 nm. Although in our experiment, we could not exclude the possibility of the involvement of other phosphatase isoforms, such as PP1, PP4, and PP5, we measured the activity of the immunoprecipitate in the presence of rather specific PP2A inhibitor Cantharidin and PP2A-siRNA and the amount of released phosphate was significantly reduced as reported in Fig. 3.

### Cell transfection with siRNA

For transfection of WB-F344 cells, we used the previously established protocol [15, 45, 46]. Briefly, cells were plated in Dulbecco’s Modified Eagle’s Medium (DMEM) (Sigma Aldrich, Milan, Italy) without antibiotics, and transfections were performed using siRNA duplexes directed against PP2A mRNA sequence (CACCAUACUCCGAGGGAAU[dT] [dT]) (Sigma Aldrich, Milan, Italy) or control siRNA-(SCR, scrambled) from Santa Cruz Biotechnology (Texas, USA). The transfections were carried using siRNA (50 nM) and Lipofectamine 2000 (Life Technologies Europe BV, Monza Italy) for 6 h, and cells were cultivated for further 24-48 h.

### Cer, C1P, and Ceramide kinase activity determination

Cer and C1P measurements were achieved as described in Bini et al. [39]. Briefly, cells were labeled with 40 μCi/ml of [^3^H] palmitate (Perkin Elmer, Connecticut, USA) for 24h in serum–free medium in the presence or absence of agonists. Cells were then washed with ice-cold calcium-free phosphate-buffered saline (PBS) and scraped into methanol and chloroform (2:1) (Sigma Aldrich, Milan, Italy). Lipids were extracted by separation of phases as described in [39]. Chloroform phases were dried, and lipids were separated by thin-layer chromatography (TLC) using silica gel 60-coated glass plates (Merck, Darmstadt, Germany). The plates were developed for 50% of their lengths with chloroform/methanol/acetic acid (9:1:1, v/v/v) and then dried. They were then developed for their full length with petroleum ether/diethylether/acetic acid (60:40:1, v/v/v) (Sigma Aldrich, Milan, Italy). The position of Cers was identified after staining with I_2_ vapor by comparison with authentic standards. Radioactivity of the samples, obtained by scraping the Cer and C1P spots from the plates, was quantified by liquid scintillation counting. CerK activity was assayed as described previously [39, 47] with some modifications.

### Measurements of cellular [^3^H]-sphingolipids

Cell monolayers were incubated with 40 μCi/ml of [^3^H]palmitate (Perkin Elmer, Connecticut, USA) complexed with 1 mg/mL fatty acid-free bovine serum albumin (Sigma Aldrich, Milan, Italy) for 24 h in serum–free medium at 37 °C. After treatment with either PCB153 or the vehicle (0.05% DMSO) for the indicated times, Cer and C1P were extracted, separated by thin-layer chromatography (Merck, Darmstadt, Germany) using chloroform/methanol/acetic acid (9:1:1, v/v/v) solution (Sigma Aldrich, Milan, Italy) as reported in [15].

### Cell cycle analysis

Cell cycle analysis was performed as in Pierucci et al. [48]. Briefly, cells treated with vehicle or 20 μM PCB153 for 24 h were collected, washed once with DPBS, and fixed with ice-cold 70% ethanol in dH_2_O to a concentration of 1 × 10^6^ to–5 × 10^6^ cells/mL. The samples were placed at least for a night at − 20 °C, then ethanol was removed, and cells were washed with DPBS + 1%BSA. After DPBS was removed, cells were stained with propidium iodide incubating them at room temperature for 30 min in the dark with the Tali® Cell Cycle Solution to a concentration of 1 × 10^5^ to 5 × 10^6^ cells/mL. 25 µl of cell suspension was transferred in the dedicated slide, and cell cycle analysis was performed using the Tali® Image-Based Cytometer.

### Statistical analysis

In immunoblot experiments, densitometric analysis of the bands was performed by ImageJ software (N.I.H., Bethesda MD, USA) (http://rsb.info.nih.gov/ij/index.html) and Quantity-One (Imaging and Analysis Software by Bio-Rad Laboratories, Hercules, CA). Band intensity was reported as relative percentage (means ± S.E.M.), obtained by calculating the ratio of specific protein on β-tubulin or β-actin intensity and normalizing to vehicle, set as 100. Mathematical analysis of electrophysiological data was performed by Pclamp 6 software (Axon Instruments, CA, USA) and Microsoft Excel (Microsoft Office 2016, Microsoft Corporation, Redmond, WA, USA). Statistical significance was determined by Student’s *t* test or one-way ANOVA and Bonferroni’s post-test for multiple comparisons. A value of *p*<0.05 was considered significant. Results are given as means ± S.E.M.

## Results

### Effect of short- and long-term PCB153 exposure on the gap junctional conductance and on Cx isoforms expression in WB-F344 cells

**Fig. 1:**
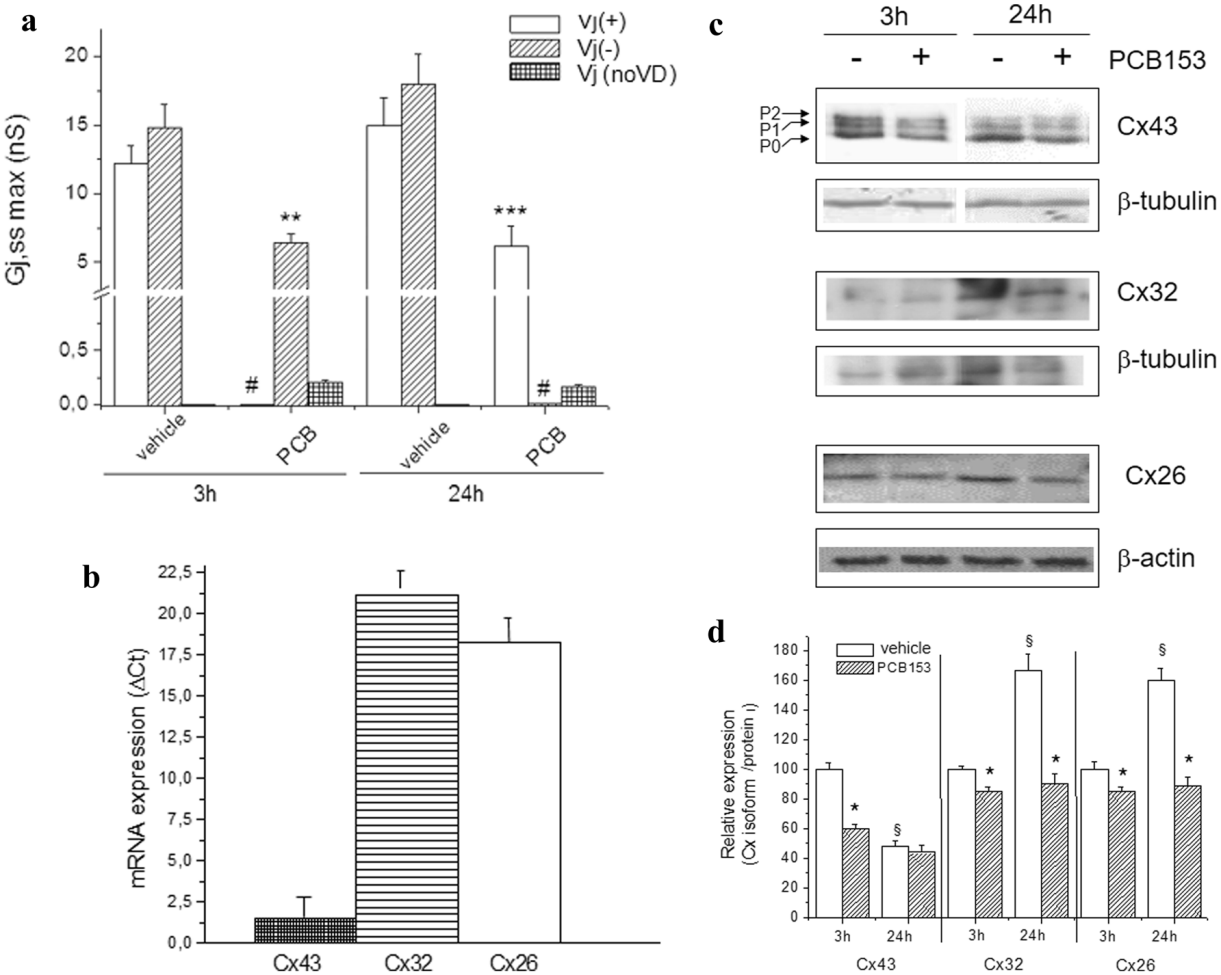
Effect of short- and long-term PCB153 exposure on the gap junctional conductance and on Cx isoforms expression in WB-F344 cells. **a** *G*_j,ss,max_ values in control condition (vehicle) and under PCB153 (3 and 24 h). Data are the mean ± SEM. The number of the investigated cells (*n*) is indicated in Table S1 for any condition. *, **, and *** indicate *p* < 0.05, *p* < 0.01, and < 0.001 of PCB153-treated cells *vs* related control (One-way ANOVA, with Bonferroni’s correction). Bar charts are related to *V*_j_ (+), *V*_j_ (−), and *V*_j_ voltage independent (indicated as *V*_j_ (no VD) in the legend). The symbol # is used when neither *V*_j_ (+) nor *V*_j_ (−) forms were observed in a treatment. All of the data for *V*_j_ (no VD) are significantly reduced (*p* < 0.001) compared to data of *V*_j_ (+) and/or *V*_j_ (−) form. **b** Real-time PCR of Cxs isoform expression. WB-F344 cells were used for total RNA preparation, Real-time PCR performed as reported in Methods section; data reported as ΔCt value (mean ± SEM of at least three independent experiments). **c** Western blotting and densitometric analysis of Cx43, Cx32, and Cx26 expression. WB-F344 cells were treated with PCB153 (20 μM) or vehicle for the indicated time. Aliquots of cell lysates (10 μg) were then subjected to SDS-PAGE, blotted onto nitrocellulose membranes, and the Cx43, Cx32, and Cx26 bands immunodetected using specific polyclonal anti-Cx43, anti-Cx32, and anti-Cx26 antibodies. **d** The percentage of reduction in band density over the control, set at 100, is reported in the graph. Data, normalized to the β-tubulin or β-actin band, are mean ± SEM of at least three independent experiments (Student’s t test, *p < 0.05 PCB153 *vs* vehicle; ^§^*p* < 0.05 24 h *vs* 3 h). A representative blot is shown

As previously reported [15], the transjunctional currents recorded in epithelial stem-like cell WB-F344 pairs usually exhibited an almost heterogeneous time course. To confirm this heterogeneity, we performed new experiments, and representative current traces (*I*_j_), recorded from two distinct cell pairs for either 3 h and for 24 h in culture are reported in Supplemental file Fig S1 (panel a, b and panel g, h, respectively) and Fig. 1a. As well, the analysis of the voltage dependence estimated by the *G*_j,ss_/*V*_j_ plot turned out to be characterized by an asymmetric behavior with a marked voltage dependence only for positive or for negative *V*_j_ polarity [15] (Supplemental file Fig.S1, panel c and i). These observations strongly indicate that GJs prevailing in this cell model are likely heterotypic. Moreover, adding PCB 153 to the culture medium caused different effects based on the time of exposure. After short-term treatment with PCB153 (20 µM, 3 h), *I*_j_ time course became almost linear especially at positive *V*_j_, suggesting a strong contribution of voltage-independent Cxs, mostly unaffected by PCB 153 (Fig. 1S, panel d, e and f). In addition, the analysis of *G*_j,ss__,__max_ showed a reduction of *V*_j_ (−) form and the disappearance of *V*_j_ ( +) form (Fig. 1a). On the other hand, after the long-term exposure with PCB153 (24 h), a different pattern of *I*_j_ and *G*_j,ss_ was observed (Fig. 1S, panels l-n). Moreover, the analysis of *G*_j,ss__,__max_ showed a reduction of *V*_j_ ( +) form and the disappearance of *V*_j_ (−) form (Fig. 1a). These findings suggest that the toxicant specifically targeted on the voltage-dependent GJs with a different time dependence (see also in supplemental file Fig. S1, Table S1).

Aiming to clarify which Cx isoforms, expressed in WB-F344 cells, were the possible targets of PCB153 action; first, we performed Real-Time PCR analysis. We found that in basal conditions, WB-F344 cells expressed Cx43, Cx32, and Cx26 mRNA at different extents (Fig. 1b). In particular, Cx43 mRNA levels were higher than those of Cx32 and Cx26, as stated by the ΔCt values. Noteworthy, in subconfluent cells, PCB153 exposure caused a significant decrease of Cx43 mRNA (ΔCt vehicle equal to 1.02 and ΔCt PCB153 equal to 1.39 mean with SEM less than 15%), without inducing any appreciable change in Cx32 and Cx26 mRNA levels (data not shown).

In control conditions, the Cx43 protein expression strongly decreased from 3h to 24h in culture, whereas the level of Cx32 and Cx26 expression increased (western blotting analysis, Fig. 1c). Interestingly, PCB153 treatment for 3 h (Fig. 1d) induced a clear down-regulation of Cx43 expression in agreement with previous literature [14, 15], but it caused only a slight decrease of Cx32 and Cx26 expression. On the other hand, prolonging PCB153 treatment until 24 h strongly reduced also Cx32 and Cx26 expression (Fig. 1d). These findings indicate that PCB153 preferentially affects Cx43 expression in short-term treatment, and its action is mainly directed to Cx32 and Cx26 during longer exposure. It is worth to note that the impairment in Cx-formed gap junction was paralleled by inhibition of cell proliferation, but no toxic effect was observed in WB-F344 cells under PCB153 exposure. In fact, we have compared the cell cycle phase distributions of vehicle- and PCB153-treated cells. In particular, the percentage of PCB153-treated cells in G_1_ phase was higher compared to that of vehicle-treated cells (80%±10 *vs* 23%±6, *n*=3), coinciding with a significant reduction in the percentage of cells in S+G_2_+M phases (18% ± 4 PCB153-treated cells *vs* 75% ± 9 vehicle-treated cells, *n*=3). This finding suggests the inhibition of G1 phase exit elicited by PCB153.

### PCB153 effects on ceramide, PP2A activity and GJ functionality

**Fig. 2:**
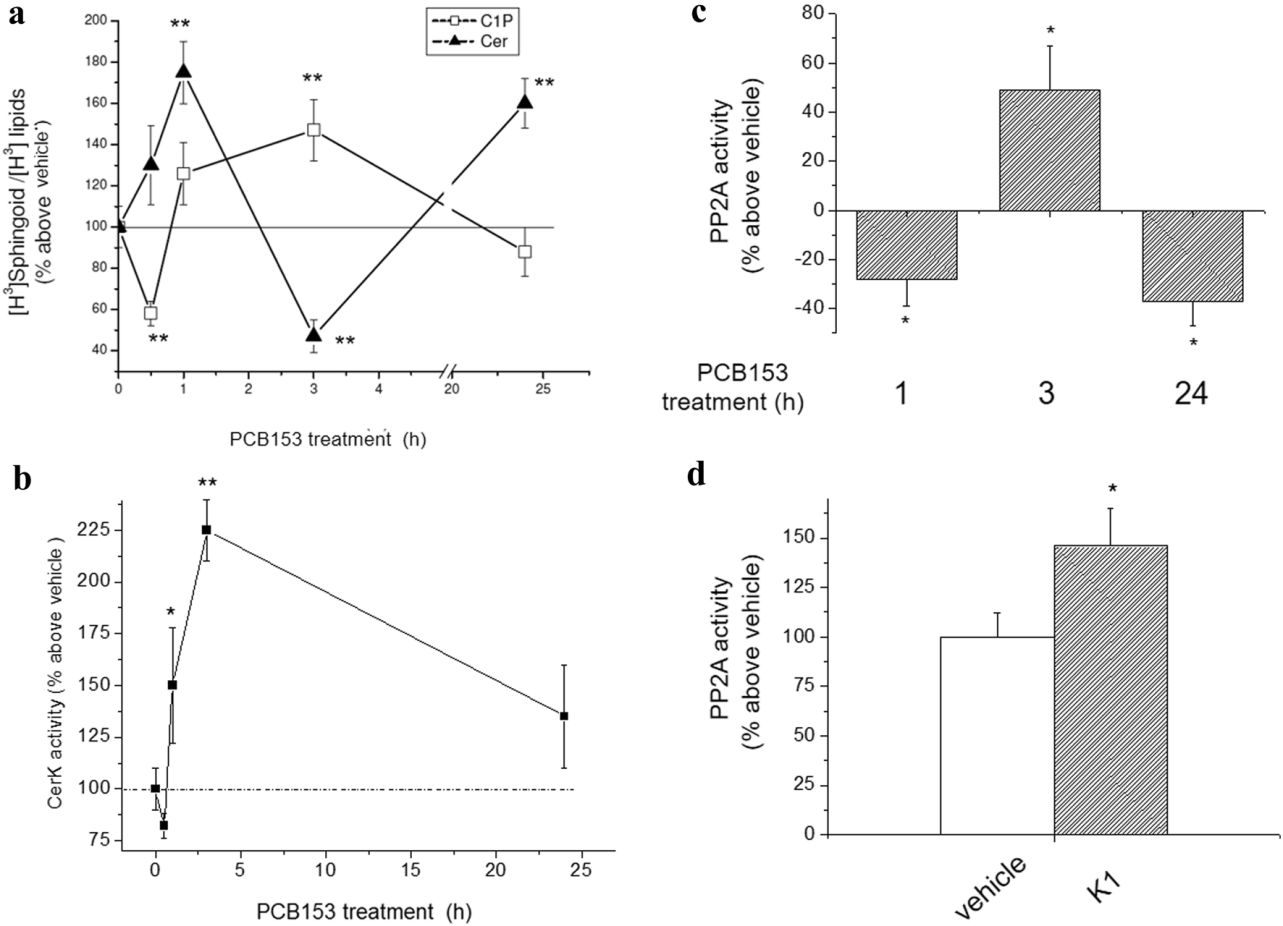
Effect of PCB153 on Ceramide 1-phosphate level, ceramide kinase activity, and PP2A activity in WB-F344 cells. **a** Cer and C1P content. Cells were incubated for 24 h with 40 μCi/ml of [^3^H]-palmitic acid for 24 h prior to the treatment with PCB153 or vehicle for the indicated time. [^3^H]Cer and [^3^H]Ceramide 1-phosphate ([^3^H]C1P) were extracted and analyzed as described in Materials and Methods. [^3^H]Cer and [^3^H]C1P content over total [^3^H]-sphingolipids are reported as means ± SEM of three independent experiments performed in duplicate (ANOVA, *p < 0.05 PCB153 *vs* vehicle). **b** Ceramide kinase activity. Enzyme activity was determined in cell lysates (40–60 μg) obtained from untreated cells (vehicle) and cells treated with PCB153 (20 μM) over the indicated times. Data are reported as mean ± SEM in at least three independent experiments (Student’s *t* test, *p < 0.05 PCB153 vs vehicle; **p < 0.01 PCB153-treated cells *vs* related control). **c** Effect of PBC153 on PP2A activity. Cells were treated for the indicated time points with 20 μM PCB153 or with vehicle. Total cell lysates (100 μg of proteins) were processed and PP2A activity determined as described in Materials and Methods. Data are reported as mean ± SEM in at least three independent experiments (Student’s *t* test, *p < 0.05 PCB153 *vs* vehicle). **d** Effect of CerK inhibitor on PP2A activity. Cells were incubated in the absence or in the presence of CerK inhibitor, K1 (10 μM). Data, reported as percentage on basal Cer activity, are mean ± SEM of at least three independent experiments performed in duplicates (Student’s *t* test, *p < 0.05 K1 *vs* vehicle)

In order to extend previous knowledge on PCB153 effect on the GJ functionality in WB-F344 cells, we then investigated in detail the possible involvement of Cer in PCB153 action. Here, we confirmed the transient alterations elicited by PCB153 in Cer/Cer1P content [15]. In particular, the level of [^3^H]Cer increased after 1 h, and decreased after 3 h of exposure to the toxicant (Fig. 2a). Similarly, the oscillations of [^3^H]C1P level with time indicated a short- and long-term effect of PCB153 (Fig. 2a).

All of these data well correlate with the kinetics of ceramide kinase (CerK) (Fig. 2b), the enzyme responsible for the phosphorylation of Cer to C1P, which showed its maximal activity within 3 h and a significant decrease at 24 h, remaining at approximately 35% above the basal level.

Since Cer is required for PP2A activation [47, 49], these findings strongly suggest that PCB153 contributes to Cer decrease and, thus, to the reduction of its positive effect on PP2A activity by modulating CerK activity in a time-dependent manner. In fact, PP2A activity was lower respect to vehicle at 1 h and higher at 3 h of toxicant exposure (Fig. 2c), being related to the changes in Cer/C1P level.

**Fig. 3:**
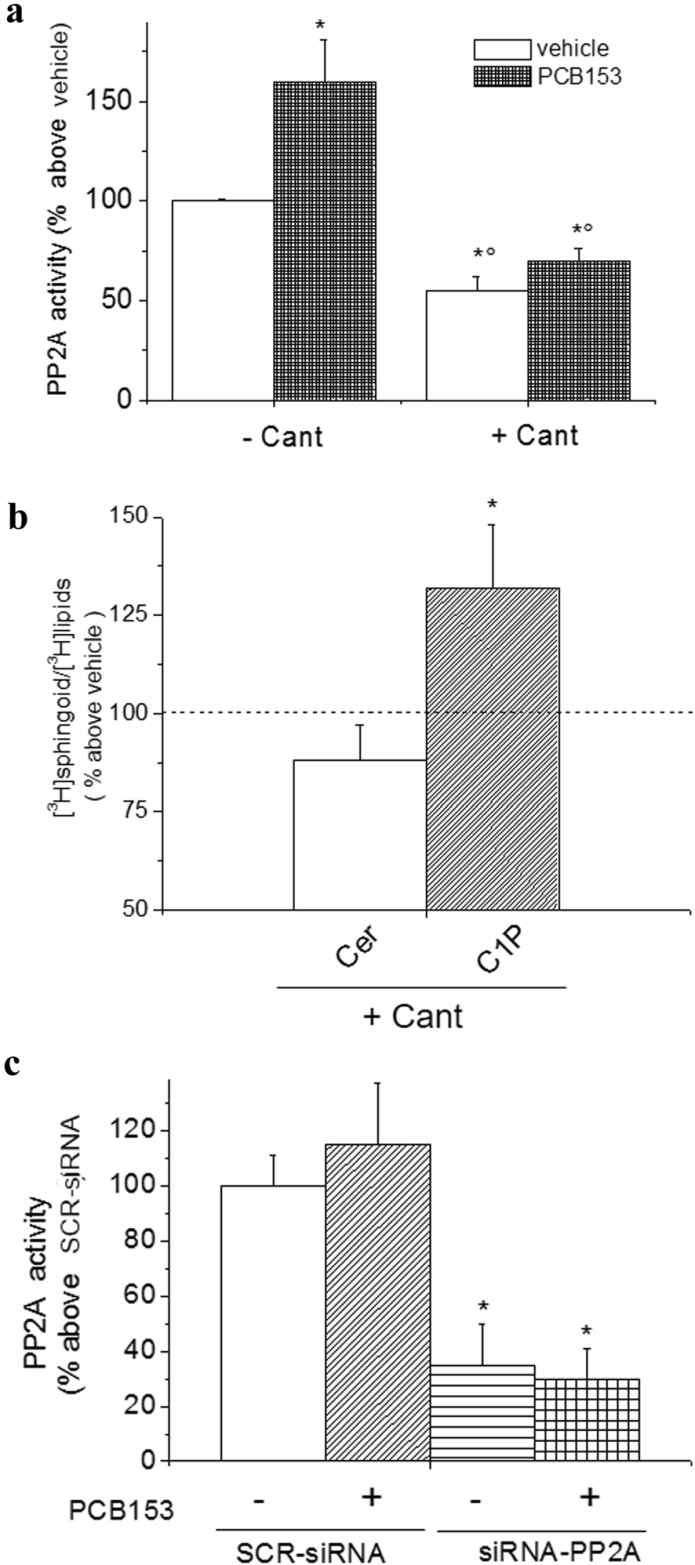
Effect of PP2A inhibition on Ceramide/ceramide 1-phosphate level and Cx43 expression/and phosphorylation in WB-F344 cells. **a** PP2A activity. Cells were treated with 3 μM Cantharidin (Cant) or vehicle for 1 h prior the exposure to vehicle or PCB153 (20 μM). PP2A activity was determined from total lysates as described in Materials and Methods. Data are reported as means ± SEM of three independent experiments performed in duplicate (ANOVA test, *p < 0.05 PCB153 *vs* vehicle. °*p* < 0.05 Cant *vs* vehicle). **b** Effect of Cantharidin (Cant) on ceramide and C1P content. Cells were incubated with 40 μCi/ml of [^3^H] palmitic acid prior to treatment with Cant or vehicle. [^3^H]Cer and [^3^H]C1P content was quantified as reported in Methods. Data are reported as means ± SEM of three independent experiments. (ANOVA test, *p < 0.05 Cant *vs* vehicle). **c** PP2A activity in PP2A-silenced cells. WB-F344 cells were treated with siRNA specific for PP2A (siRNA-PP2A) or scrambled siRNA (SCR-siRNA) and treated with vehicle or PCB153. PP2A activity was determined from total lysates as described in Materials and Methods. Data are reported as means ± SEM of at least three independent experiments (Student t test, *p < 0.05 siRNA-PP2A *vs* SCR-siRNA)

To further support the relation between CerK and PP2A activity, we performed additional experiments by using pharmacological as well as gene silencing approaches (Figs. 2d and 3). In the presence of the rather specific inhibitor of CerK, K1, PP2A activity resulted enhanced (Fig. 2d). Moreover, the pharmacological inhibition of PP2A by Cantharidin (Cant), in addition to reducing the activity of PP2A (Fig. 3a), enhanced C1P level, with a slight but not significant effect on Cer content (Fig. 3b). Moreover, the gene silencing of PP2A strongly reduced PP2A activity either in the absence or in the presence of PCB153 (Fig. 3c). These findings provide the first evidence of an inverse correlation between CerK and PP2A activity in liver epithelial stem-like cells.

**Fig. 4:**
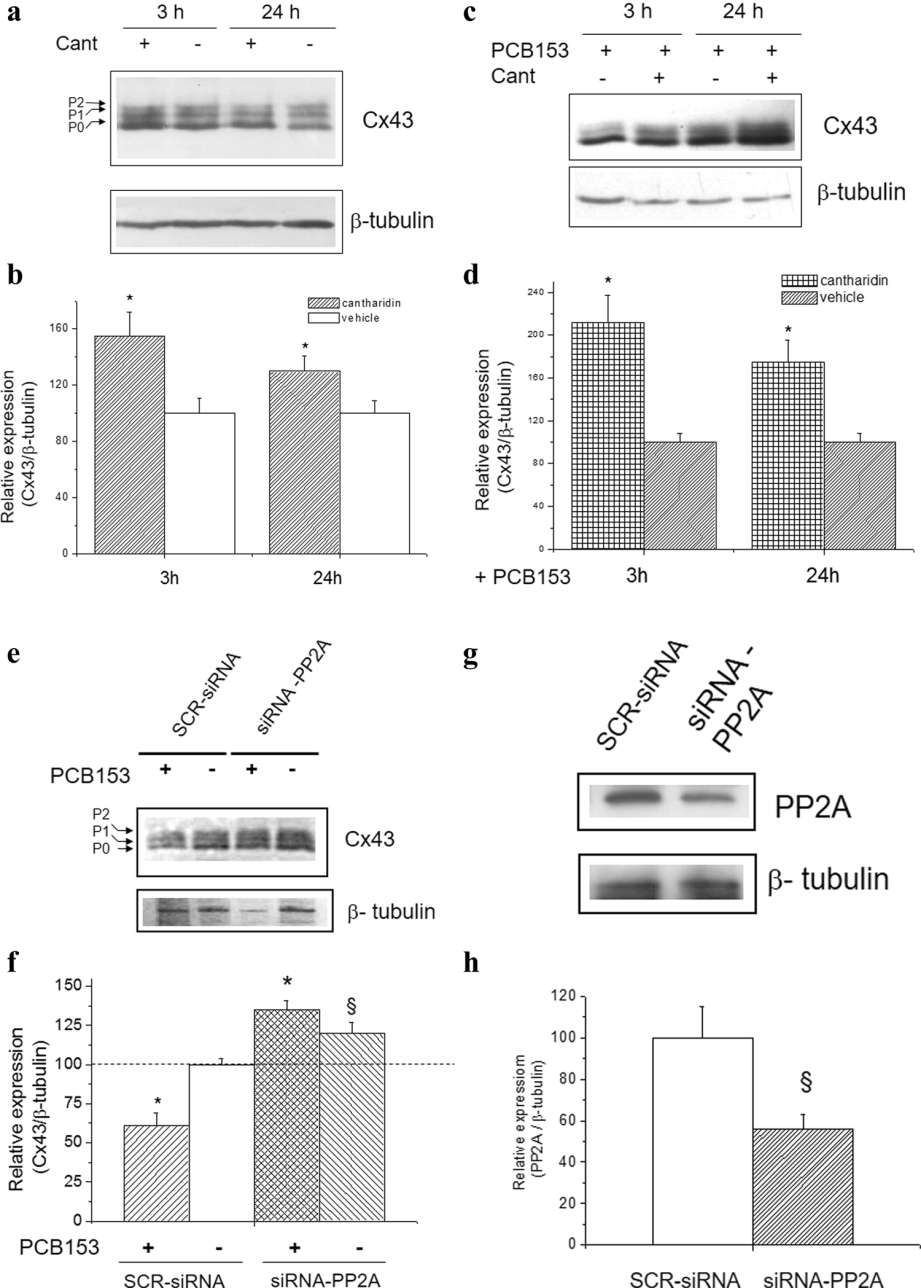
Effect of PP2A inhibition and PC153 on Cx43 expression and protein phosphorylation. Western blotting and densitometric analysis of Cx43 and PP2A expression. WB-F344 cells were treated with 3 μM Cantharidin (Cant) or vehicle (0.02% DMSO) (**a**, **b**) and/or with PCB153 (**c**, **d**) and transfected with siRNA specific for PP2A (siRNA-PP2A) or scrambled siRNA (SCR-siRNA) (**e**–**h**). Aliquots of cell lysates (15 μg) were processed as in Materials and Methods. Cx43, PP2A, and β-tubulin band intensity were determined, and densitometric data are reported in the graphs (Student’s *t* test, in a: *p < 0.05 Cant *vs* vehicle; in c:*p < 0.05 PCB153 + Cant *vs* PCB153; in e: *p < 0.05 PCB153 *vs* vehicle (^§^*p* < 0.05 siRNA-PP2A *vs* SCR-siRNA) and in g (^§^*p* < 0.05 siRNA-PP2A *vs* SCR-siRNA). Representative blots are shown

Among the voltage-sensitive isoforms expressed in WB-F344 cells, Cx43 is the most abundant and the most regulated by phosphorylation [50]. Thus, we further studied the potential role of PP2A in mediating its expression (Fig. 4). In particular, the basal level of Cx43 expression appeared increased either at 3 h or at 24 h in the presence of the PP2A inhibitor, Cant (Fig. 4a and b), as well as of siRNA-PP2A (Fig. 4e and f). On the other hand, in the presence of PCB153 and PP2A inhibitor, the level of Cx43 expression was even higher than that observed in the presence of PCB153 alone (Fig. 4c and d). The silencing of PP2A by siRNA-PP2A confirmed the ability of the PP2A inhibitor, Cant, to revert the GJ protein down-regulation elicited by PCB153 (Fig. 4e and f).

These new results indicate that PCB153, modulating Cer/C1P axis, affects PP2A activity and, in turn, Cx43 expression and its phosphorylation status.

**Fig. 5:**
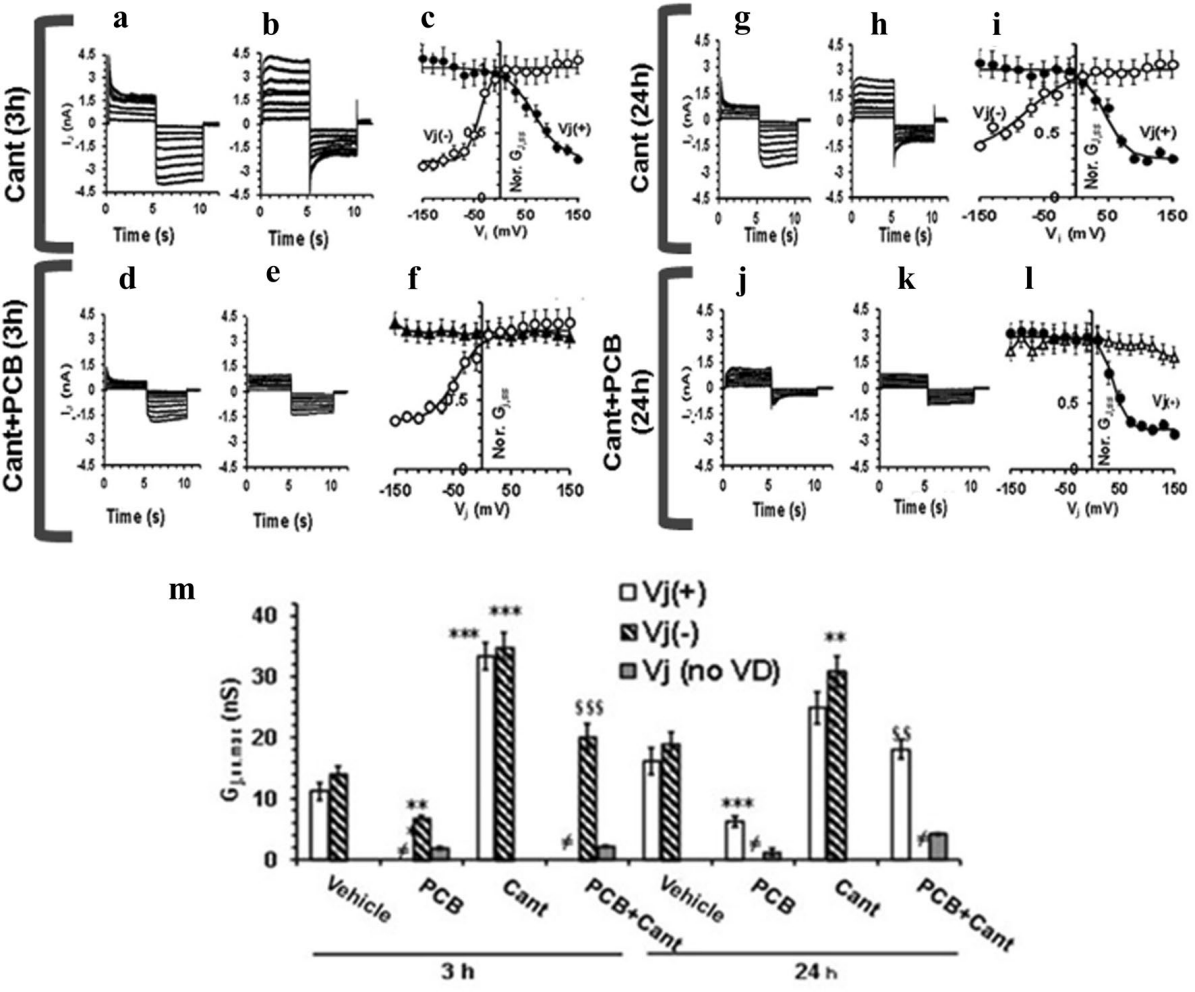
Effect of Cantharidin treatment on transjunctional currents, *I*_j_, time course (in nA) and on *G*_j,ss_ (in nS) in WB-F344 cell pairs. Representative *I*_j_ time courses recorded in WB-F344 cell pairs treated with Cantharidin alone (Cant) for 3 h (**a**, **b**) and for 24 h (**g**, **h**) or with PCB153 added in the presence of Cantharidin (Cant + PCB) for 3 h (**d**, **e**) and 24 h (**j**, **k**). The current traces recorded from various cell pairs are here shown for each experimental condition to highlight the heterogeneous behavior of the distinct cell pairs: each panel shows a family of *I*_j_ traces recorded from a different cell pair (note the differences between **a** and **b**, **d** and **e**, **g** and **h**, **j** and **k**). **c**, **i** Normalized *G*_j,ss_/*V*_j_ plots related to all of the experiments done in cantharidin-treated cells for 3 h (**c**) and 24 h (**i**). *G*_j,ss,max_ values in Cant are the mean values deriving from the cells with *V*_j_ (+) and *V*_j_ (−) asymmetric curves (filled and open circles, respectively). **f**, **l** Normalized *G*_j,ss_/*V*_j_ plots related to all of the experiments done in Cant + PCB for 3 h (**f**) and 24 h (**l**). *G*_j,ss,max_ in Cant + PCB-treated cells could be voltage dependent at negative or positive Vj (open and filled circles) or voltage independent for any *V*_j_ values (triangles). Superimposed lines represent the fit of a single Boltzmann function to the *G*_j,ss_/*V*_j_ plot either with inactivating *V*_j_, at *V*_j_ (+), or *V*_j_ (−). **m** *G*_j,ss,max_ values in control condition (vehicle), PCB153 (PCB), Cantharidin (Cant), and under PCB153 + Cantharidin (PCB + Cant) treatment for 3 and 24 h. The number of the investigated cells (n) is indicated in Table S1. ** and *** indicate *p* < 0.01 and *p* < 0.001 *vs* vehicle. §§ and §§§ indicate *p* < 0.01 and *p* < 0.001 *vs* PCB153 alone (One-way ANOVA, with Bonferroni’s correction). Bar charts are related to *V*_j_ (+), *V*_j_ (−), and *V*_j_ voltage independent (indicated as *V*_j_ (no VD) in the legend). The symbol ≠ is used when neither *V*_j_ (+) nor *V*_j_ (−) forms were observed in a treatment. Data are the mean ± SEM. Values resulting from the Boltzmann fit are listed in Table S1.

To evaluate the effect of these treatments on GJ functionality, we achieved the electrophysiological analysis of the currents flowing through the GJs. The results are displayed in Fig. 5, where we show different current traces recorded from two distinct cell pairs for each experimental condition: the records in panel a are obtained from a cell pair different from b, d different from e, g different from h and j different from k, to highlight the heterogeneity of the cell pairs. Cant alone induced time-dependent changes compared to control cells: the *I*_j_ time course and voltage dependence evaluated at 3 h (Fig. 5c) and 24 h (Fig. 5i) showed a clear voltage dependence only at *V*_j_ (−) or only at *V*_j_ ( +), as in vehicle (Fig. S1 in supplemental file). However, Cant induced a significant increase of *G*_j,ss,max_ compared to vehicle, either at short- or long-term treatment (Fig. 5m), suggesting that the inhibition of PP2A could somehow improve GJIC.

Conversely, when PCB153 was added in the presence of Cant, *I*_j_ showed a similar time course but an increased amplitude (Fig. 5d, e, j and k) compared to what was observed in cell pairs treated with PCB153 alone (Fig. 1s), both for 3 h and 24 h. The *G*j_,ss/_*V*j plots shown in Fig. 5f, l state the analysis of the voltage dependence. Notably, PCB153 added in the concomitant presence of Cant was less effective in reducing *G*_j,ss,max_ than PCB153 alone, at any time point of exposure (Fig. 5m), especially considering the effect on voltage-dependent GJ.

Overall, these novel results indicate that PP2A inactivation determines the enhancement of the GJ functionality paralleled by the upregulation of both the voltage-dependent and -independent Cxs. On the other hand, PCB153 treatment made in the concomitant presence of Cant, was no more able to hinder the GJ functionality, suggesting that PP2A counteracts the toxicant effects acting preferentially on the voltage-dependent Cxs (Fig. 5; supplemental file Table S1).

### Role of ceramide on GJ and Cx43 expression

**Fig. 6:**
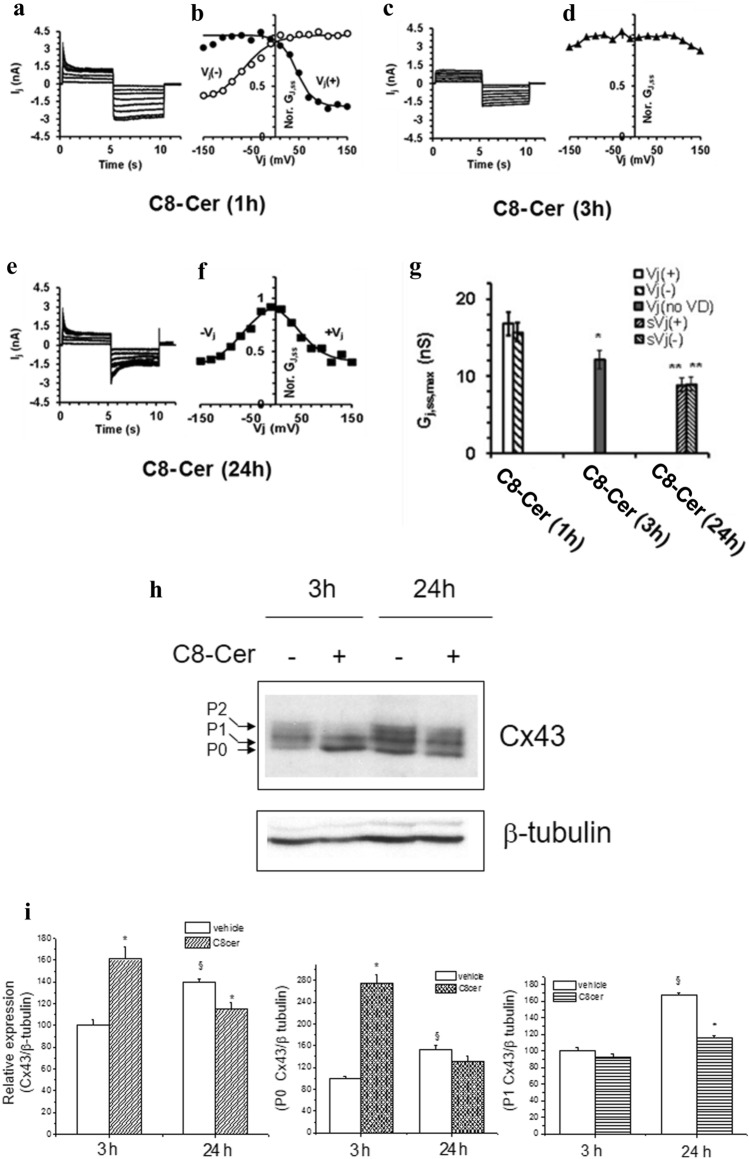
Time-dependent effect of C8-ceramide on *I*_j_ time course (in nA), *G*_j,ss_/*V*_j_ plot, *G*_j,ss,max_, and Cx43 expression in WB-F344 cells. Typical *I*_j_ families evaluated in WB-F344 cell pairs 1, 3, and 24 h after C8-ceramide (C8-Cer) treatment (panel **a**, **c** and **e**, respectively) and related normalized *G*_j,ss_/*V*_j_ plots (panel **b**, **d,** and **f**, respectively). The superimposed continuous lines through the data points represent the fit with a Boltzmann function. **b** Asymmetric *G*_j,ss_/*V*_j_ plots resulting after 1 h of C8-Cer treatment (filled and open circles indicate *V*_j_ (+) and *V*_j_ (−), respectively). **d** C8-Cer treatment up to 3 h alters the *G*_j,ss,max_ voltage dependence (filled up triangles). **e** and **f** After 24 h of C8-Cer treatment, the *I*_j_ time course become symmetrical as well as the *G*_j,ss_/*V*_j_ plot (**f**, filled squares). **g** Comparison of *G*_j,ss,max_ values (nS) obtained under C8-Cer treatment for different times; the symbols s*V*_j_(+) and s*V*_j_(−) represent the positive and negative voltage dependence observed in symmetrical GJ after 24 h. Data are the mean ± SEM. Boltzmann parameters, statistical significance, and number of investigated cell pairs are in Table S2 (One-way ANOVA, with Bonferroni’s correction). **h** Western blotting and densitometric analysis of Cx43 expression. WB-F344 cells were treated with C8-Cer (8 μM) or vehicle for the indicated time. Aliquots of cell lysates (10 μg) were then subjected to SDS-PAGE, blotted onto nitrocellulose membranes, and the Cx43 bands immunodetected using specific polyclonal anti-Cx43 antibodies. **i** The percentage of reduction in band density, over the control set at 100 is reported in the graph. Data, normalized to the β-tubulin band, are mean ± SEM of at least three independent experiments (Student’s *t* test, *p < 0.05). A representative blot is shown.

In order to elucidate the role of Cer on the signaling cascade promoted by PCB153, we treated the cells with C8-ceramide (C8-Cer, 8 µM), a cell permeable synthetic analog of Cer. Our results indicate that C8-Cer operated on GJIC functionality in a time-dependent manner (Fig. 6). In particular, after 1h, both the *I*_j_ time course and the *G*_j,ss_/*V*_j_ relation were asymmetric, showing only the *V*_j_ (−) or *V*_j_ (+) form, as those observed in control (Supplemental file Table S2). In the short-term treatment (3h), these features drastically changed, displaying an almost voltage-independent shape (Fig. 6c and d). These findings suggest the involvement of homotypic GJs made of two equal hemichannels likely formed by scarcely voltage-dependent Cx, like Cx26. C8-Cer effect was similar to that observed in WB-F344 cells treated with sphingosine kinase inhibitor (iSK) [15].

In the long-term treatment (24h), the *I*_j_ time course and the *G*_j,ss_/*V*_j_ plot changed again, being almost symmetrical and essentially voltage dependent (Fig. 6e, f and g), indicating that only homotypic GJs formed by voltage-dependent Cxs remain functional under long-term C8-Cer treatment.

The analysis of the phosphorylatable Cx isoform expression, Cx43, showed that C8-Cer treatment for 3 h increased its overall amount, affecting the P0 form, without affecting the level of the phosphorylated P1 form. These data indicate that short-term treatment with C8-Cer drastically affects the GJs, decreasing the voltage-dependent GJ functionality, without altering the phosphorylation status of Cx43. In contrast, at long-term C8-Cer exposure (24 h), the overall Cx43 expression significantly decreased as well as the levels of the phosphorylated form P1 (Fig. 6h and i).

Since only homotypic voltage-dependent GJs remain functional in these experimental conditions, we cannot exclude the presence of other highly voltage-dependent Cxs. This point deserves further investigation in a future study.

## Discussion

PCBs are a large family of environmental pollutants that are both phenobarbital (PB)-like inducers of hepatic drug metabolism and tumor promoters in rodent liver [51]. The tumor promotion involves many non-genomic signals and, among these, the inhibition of GJIC is certainly critical. Previous in vitro studies on stem-like epithelial cell line WB-F344 showed that the nonplanar PCB153 is a strong inhibitor of GJIC [11]. Different signaling pathways are involved in the toxicant-induced down-regulation of GJIC, such as those downstream to phospholipase C (PLC) or sphingomyelinase and proto-oncogene tyrosine*-*protein kinase (Src) [11]. In addition, our research group recently showed that PCB153 exposure affects the sphingolipid metabolism reducing the synthesis of the pro-survival metabolite S1P [15]. Of note, the balance between S1P and its lipid precursor, Cer, known as the “sphingolipid rheostat,” has been found to be crucially involved in pathological conditions, including liver diseases and tumorigenesis [52–54]. Cer is also believed to be a potent activator of the PP2A [46], the ubiquitously expressed serine/threonine phosphatase that plays a key role in many cellular events [30, 31], including cell cycle progression, DNA replication, signal transduction, cell proliferation, and cytoskeleton dynamics [32] and, depending on the cell context, acts either as a tumor suppressor or as a tumor promotional factor [31]. A number of studies actually report that PP2A is positively regulated by Cer [33, 34]. The involvement of bioactive SLs in the regulation of the GJs has been reported in in vitro models of normal cells, such as astrocytes and skeletal muscle cells [35, 36], in cancer cells [37] and in vivo in sphingomyelin-fed animals.

In the present research, we demonstrated that PCB153 is able to affect GJIC through a Cer-mediated signaling pathway. In particular, we highlight some outcomes coming from a combined electrophysiological and biochemical analysis based on current records, *G*_*j*_*/V*_j_ plot analysis and protein expression determination. In this view, the electrophysiological experiments helped us to postulate the kind of GJIC involved. We should remark that, based on the positive or negative transmembrane voltages (*V*_j_) applied, the heterotypic GJs show an asymmetrical dependence of the conductance (*G*j) on transjunctional voltages [19], as estimated by the *G*_j_*/V*_j_ plot. In contrast, homotypic GJs usually show a symmetric voltage dependence of the conductance, but this voltage sensitivity differs based on the composition of Cx isoforms. With this in mind, one of our first result is that WB-F344 cells mainly express heterotypic GJs that can be formed by Cx43, Cx32 and Cx26 isoforms and PCB153 affects them in a time-dependent manner. How the different Cx isoforms dock to form functional heteromeric and heterotypic GJs depends on their mutual compatibility [55].

The second main outcome of this study is the inverse correlation between PP2A activity and Cx43 expression: indeed, in some cell pairs, inhibiting PP2A increases Cx43 expression and enhances the occurrence of heterotypic GJICs formed by both voltage-dependent and voltage-independent Cxs.

The third interesting point of this study is that PCB153 preferentially down-regulates the function of the voltage-dependent Cxs-formed GJIC. Notably, we previously found that the voltage-dependent Cxs-formed GJIC were completely blocked by the inhibition of SphK/S1P axis [15], being the observed GJIC completely or prevalently voltage independent (Fig. S1). Here, we obtained similar data after C8-Cer treatment, a cell permeable analog of Cer, but only after 3 h, indicating that the C8-Cer effect is a time-dependent event, which mimics the effect of SphK inhibition at this short-term point. These latter considerations, together with the here reported down-regulation of CerK elicited by PCB153, indicate that the toxicant influence on voltage-dependent and -independent Cxs depends on SphK/S1P and on Cer/PP2A axis in a time-dependent manner. In particular, it depends on Cer increase and, in turn, on PP2A activation.

Overall, our findings confirm the GJIC modulation operated by Cer reported by Park et al. [56] and offer new interesting evidence that GJs made of different Cxs isoforms are transiently modulated. Indeed, we here demonstrated that only homotypic GJs remain functional under a short-term treatment with C8-Cer and these possibly consisted of the voltage-independent Cx (likely Cx26). In contrast, after 24 h, C8-Cer causes the activation of the voltage dependent and the reduction of the voltage-independent Cxs forming the GJs. The *I*_j_/*V*_j_ relation of GJIC was indeed symmetrical, denoting that only homotypic GJs, made by voltage-dependent Cxs, were functional. The observed resistance of the voltage-independent Cx to PCB153, likely represented by Cx26, underlines the crucial role of this Cx isoform in guaranteeing a basal minimal intercellular communication when the cells are exposed to the toxicant, even if PCB153 exposure slightly reduces its expression.

Notably, Neveu et al. [57] and Matesic et al. [58] previously reported the presence of Cx26 in WB-F344 cells. They also demonstrated the alterations of Cx26 and Cx43 expression in proliferating cells (i.e., hepatocytes and non-parenchymal liver cell types), suggesting that changes in Cx26 expression may be correlated to a higher proliferation rate. On this line, another study reported that Cx26 drives self-renewal in triple-negative breast cancer [59] by interacting with focal adhesion kinase and NANOG, indicating a role in targeting and compromising cancer stem cell maintenance. Furthermore, mutations in Cx26 gene (GJB2) in humans, in addition to cause hearing loss [60], lead to the development of skin and primary breast tumors, suggesting again that the alterations of Cx26 functionality may predispose to cancer [61, 62]. In keeping with these assumptions, we can speculate that Cx26 target of Cer signaling may have a key role for explaining the long-term non-genomic action of PCB153 as a possible tumor promoter. Although the role of Cer/CerK axis in hepatocellular carcinoma (HCC) is unclear, a dysregulation of Cer metabolism has been reported in HCC progression [63, 64], in particular of Cer synthases and ceramidases.

## Conclusions

Our findings provide novel evidence that (1) the voltage-dependent and-independent Cx-formed GJs are expressed in liver stem-like epithelial cells; (2) the non-dioxin-like toxicant differently affects the voltage-independent and -dependent Cx-formed GJs (graphical abstract Fig S2, Supplemental file) the environmental toxicant PCB153 modulates GJs through Cer/CerK/PP2A axis.

These findings open new perspectives in the development of preventive approaches against the toxic effects of the NDL compounds.

## Supplementary Information

Below is the link to the electronic supplementary material.

Supplementary file1 (DOCX 127 KB)

Supplementary file2 (DOCX 18 KB)

Supplementary file3 (DOCX 21 KB)

Supplementary file4 (PPTX 80 KB)

## Data Availability

The dataset(s) supporting the conclusions of this article is (are) included within the article (and its additional file(s)).

## Abbreviations

PCBs: Polychlorinated Biphenyls
PCB153: 2,2′,4,4′,5,5′-Hexachlorobiphenyl
GJIC: Gap Junction Intercellular Communication
Cx: Connexin
GJ: Gap junctions
SL: Sphingolipids
S1P: Sphingosine 1-Phosphate
Cer: Ceramide
CerK: Ceramide Kinase
C1P: Ceramide 1-Phosphate
PP2A: Protein Phosphatase 2A
C8-Cer: C8-ceramide
K1: Ceramide kinase inhibitor
Cant: Cantharidin
AhR: Aryl hydrocarbon Receptor
