# Supplementary material for: Ceramide/protein phosphatase 2A axis is engaged in gap junction impairment elicited by PCB153 in liver stem-like progenitor cells
Source: Mol Cell Biochem. 2021 Apr 10;476(8):3111–26. doi: 10.1007/s11010-021-04135-z (PMC8263450; doi:10.1007/s11010-021-04135-z)
Supplement: Supplementary file 1 — Supplementary file1 (DOCX 127 KB) [file 11010_2021_4135_MOESM1_ESM.docx]

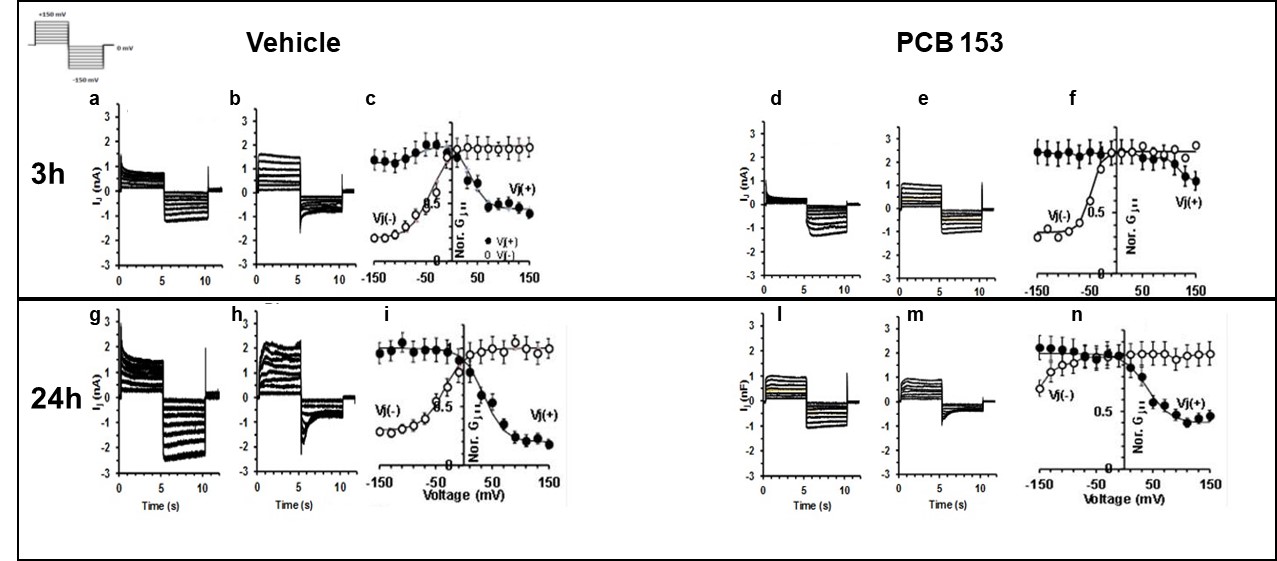


**Fig. S1. Effect of PCB153 exposure on the time course of transjunctional currents and on the gap junctional conductance voltage-dependence in WB-F344 cells.**

Time course of typical Ij current traces recorded in two distinct control cell pairs (vehicle, panels a and b) or in two different PCB153-treated cell pairs for 3h (d and e), evoked in response to the bipolar pulse protocol shown in the inset above panel a. In control conditions Ij is strongly asymmetrical being voltage dependent only for negative or positive Vj. In PCB153-treated cells (3 h) Ij is voltage dependent at negative Vj and voltage independent at positive Vj. For clarity only the traces elicited at +/- 10, 30, 50, 70, 90, 110, 130 and 150 mV are presented. The voltage-dependence of the Normalized gap junctional conductance estimated from all of the experiments done (3h) is shown for the control condition (c) and for the PCB 153 treatment (f). The conductance evaluated at the steady-state (G_j,ss_) in control cells (vehicle, c) shows asymmetrical voltage dependence with Vj(-) or Vj(+) form (open and filled circles, respectively).

Representative time course of Ij current traces recorded in two distinct control cell pairs (g and h) or in two different PCB153-treated cell pairs for 24h (l and m).

he voltage-dependence of the Normalized gap junctional conductance estimated from all of the experiments done (24h) is shown for the control condition (i) and for the PCB 153 treatment (n).

Data are the mean ± SEM. Values resulting from the fit of a single Boltzmann function to the normalized G_j,ss_/Vj plots and the number of the investigated cells are indicated in Table 1S with the statistical significance.
