# Supplementary material for: Ceramide/protein phosphatase 2A axis is engaged in gap junction impairment elicited by PCB153 in liver stem-like progenitor cells
Source: Mol Cell Biochem. 2021 Apr 10;476(8):3111–26. doi: 10.1007/s11010-021-04135-z (PMC8263450; doi:10.1007/s11010-021-04135-z)
Supplement: Supplementary file 2 — Supplementary file2 (DOCX 18 KB) [file 11010_2021_4135_MOESM2_ESM.docx]

**Table S1. Boltzmann Parameters for Gap Junctional conductance (*G*_j,ss_) after 3 h and 24 h in control (vehicle), under PCB, Cant, and Cant+PCB treatments.**

|  |  | **A (mV^-1^)** | **V_0_ (mV)** | **G_min_** | **G_j,ss,max_ (nS)** | **N** |
| --- | --- | --- | --- | --- | --- | --- |
| Vehicle  (3h) | V_j_(+)  V_j_(-) | 0.06±4.10^-3^  0.05±3 10^-3^&& | 35.1± 4.6  -41.0±4.3& | 0.5±0.03  0.2±0.04&&& | 11.2±1.4  14.1±1.2&& | 8  9 |
| Vehicle (24h) | V_j_(+)  V_j_(-) | 0.06±3 10^-3^  0.06±3 10^-3^& | 35.1±5.4  -35.3±4.2§ | 0.2±0.03&&§§  0.3±0.05&§ | 16.1±2.2§§  18.9±2.0&§§ | 9  8 |
| PCB  (3h) | V_j_(±)  V_j_(-) | No VD  0.09±4 10^-3^*** | No VD  -48.3±4.2** | No VD  0.3±0.05** | 1.9±0.2  6.9±0.4*** | 7  8 |
| PCB  (24h) | V_j_(±)  V_j_(+) | No VD  0.05±4 10^-3^ §§§* | No VD  39.8±5.4§§ | No VD  0.4 ± 0.03§§*** | 1.2±0.2§§  6.2±0.4§*** | 9  8 |
| Cant  (3h) | V_j_(+)  V_j_(-) | 0.04±4 10^-3^***  0.09±4 10^-3^&&&** | 64.8±6.2***  -39.1±4.1&& | 0.3±0.03**  0.3±0.04** | 33.4±2.2***  34.9±2.3** | 7  9 |
| Cant  (24h) | V_j_(+)  V_j_(-) | 0.06±4 10^-3^ §*  0.03± 4 10^-3^&&§§§** | 55.8 ± 5.2§**  -76.2±3.9&&§§§** | 0.3±0.04**  0.4 ± 0.04&§* | 24.9±2.6***  30.9±2.5&§*** | 9  7 |
| Cant+PCB (3 h) | V_j_(±)  V_j_(-) | No VD  0.05±4 10^-3^** | No VD  -40.9±4.2 | No VD  0.4±0.05** | 2.2±0.2  20.2±2.0 | 9  8 |
| Cant+PCB (24 h) | V_j_(±)  V_j_(+) | No VD  0.08±4 10^-3^** | No VD  38.9±5.0 | No VD  0.3±0.06 | 4.2±0.5§§  18.1±1.6 | 8  7 |

Boltzmann parameters related to the steady-state gap junctional conductance (*G*_j,ss_**)** evaluated in control (Vehicle), PCB, Cant, Cant+PCB, treated cell-pairs for 3 and 24 h. Data are obtained by fitting a Boltzmann function to the normalized steady-state conductance versus voltage plot. *G*_j,ss,max_, evaluated at -10/+10 mV represents the maximal value of *G*_j,ss_. *G*_min_ data are normalized to *G*_j,ss,max_. V_j_(+) and V_j_(-) indicate the type of asymmetric *I*_j_ form, and V_j_(±) the voltage independent form (No VD). Statistical significance is calculated by one-way ANOVA with the Bonferroni’s correction: &, && and &&& indicate P < 0.05, 0.01 and 0.001 for *V*_j_(-) form *vs*. the related *V*_j_ (+) form in the same cell pair at 3 or 24 h; §, §§ and §§§ are P < 0.05, 0.01 and 0.001 for data at 24 h *vs*. the corresponding data at 3h;*, ** and *** indicate P< 0.05, 0.01 and 0.001 for the treated cell pairs *vs.* the corresponding control values at 3 or 24 h. Data are mean ± SEM. N indicates the number of cell-pairs investigated.

**Table S2. Boltzmann Parameters for Gap Junctional conductance (*G*_j,ss_) under C8-Cer treatment.**

| **C8-Cer Treatment** |  | **A (mV^-1^)** | **V_0_ (mV)** | **G_min_** | **G_j,ss,max_ (nS)** | **N** |
| --- | --- | --- | --- | --- | --- | --- |
| 1 h | V_j_(+)  V_j_(-) | 0.04±4.10^-3^  0.03±3.10^-3^& | 45.1±4.6  -65.0±5.3&& | 0.32±0.03  0.35±0.05 | 16.8±1.5  15.7±1.2 | 5  7 |
| 3 h | V_j_ (±) | No VD | No VD | No VD | 12.1±1.0 * | 7 |
| 24 h | sV_j_(+)  sV_j_(-) | 0.06 ± 4.10^-3^&##  0.03±4.10^-3^&&** | 43.2±4.2  -63.2±4.2&& | 0.38±0.05  0.36±0.05 | 8.9±0.7**  9.0±0.7** | 6 |

Boltzmann parameters related to the steady-state gap junctional conductance (*G*_j,ss_**)** evaluated in C8-ceramide treated cell-pairs for 1, 3 and 24 h. Data are obtained by fitting a Boltzmann function to the normalized steady-state conductance **(***G*_j_**_,ss_**) versus voltage plot. *G*_j,ss,max_, evaluated at -10/+10 mV represents the maximal value of *G*_J,ss_ (*G*_J,ss,max_). *G*_min_ data are normalized to *G*_J,ss,max_. After 1 h of C8-ceramide treatment *G*_j,ss_ is voltage-dependent in some cell pairs only for positive *V*_j_ values, *V*_j_(+) form, and in some others only for negative *V*_j_ values, *V*_j_(-) form. After 3 h of C8-ceramide treatment *G*_j,ss,max_ values distribution was voltage-independent for any transjunctional voltage (indicated as V_j_ (±) and ‘No VD’). After 24 h the plots are symmetric and the Vj at positive and negative voltage are indicated as sV_j_(+) and sV_j_(-), respectively (‘s’ denotes the symmetrical form). Statistical significance is calculated by one-way ANOVA with the Bonferroni’s correction. & and && indicate P < 0.05 and 0.01 for V_j_(-) versus V_j_(+) in C8-Cer 1 h and sV_j_(-) versus sV_j_(+) in C8-Cer 24 h; * and ** indicate C8-Cer at 3 and 24 h compared to 1 h; ## P < 0.01 for C8-Cer 24 h compared to Cer 1 h. Data are mean ± SEM. N indicates the number of cell-pairs investigated.
