# Supplementary material for: Ceramide/protein phosphatase 2A axis is engaged in gap junction impairment elicited by PCB153 in liver stem-like progenitor cells
Source: Mol Cell Biochem. 2021 Apr 10;476(8):3111–26. doi: 10.1007/s11010-021-04135-z (PMC8263450; doi:10.1007/s11010-021-04135-z)
Supplement: Supplementary file 3 — Supplementary file3 (DOCX 21 KB) [file 11010_2021_4135_MOESM3_ESM.docx]

**SUPPLEMENTARY FILES**

**Methods**

**Cell culture and chemical treatments**

Rat liver epithelial stem-like cell WB-F344 cells, non-tumorigenic epithelial cell line, kindly provided by Dr. JE. Trosko (Michigan State University, East Lansing, MI, USA) (Trosko et al., 1984), were cultured as reported in (Pierucci et al., 2017; Šimečková et al., 2009a). S1P and Cantharidin (Cant), a rather specific inhibitor of PP2A (Tocris, Bristol, UK) (Kim et al., 2013) were dissolved in DMSO (less than 0.1% v/v) or PBS. Cells were treated with 20 µM PCB153 (a non-cytotoxic concentration) for the indicated time. The effect of C8-ceramide (8 µM) (Sigma Aldrich, Italy), the cell permeable synthetic analogues of ceramide, was evaluated after 1, 3 and 24 h in culture.

**Electrophysiological records**

The functionality of the gap junction channels between WB-F344 cell pairs was tested by using the dual whole-cell patch clamp in voltage-clamp conditions, as previously described (Valiunas et al., 2000; Formigli et al., 2005; Squecco et al., 2006; Gonzàles et al., 2007; Meacci et al., 2010; Pierucci et al., 2017). Coverslips with adherent cells were placed in the recording chamber on the stage of an inverted microscope (Nikon Eclipse TE 2000). The cells were superfused with a normal Tyrode bath solution containing (in mM) 140 NaCl, 5.4 KCl, 1.8 CaCl_2_, 1.2 MgCl_2_, 10 D-glucose, and 5 HEPES. The patch pipettes were filled with a filling pipette solution containing (in mM) 150 CsBr, 5 MgCl_2_, 10 EGTA, and 10 HEPES filtered through 0.22-µm pores. The pH was set to 7.4 with NaOH and to 7.2 with tetraethylammonium-OH for bath and pipette solution, respectively. By using a micropipette vertical puller (Narishige PC-10; Narishige, Kyoto, Japan), we pulled the patch pipettes from borosilicate glass (GC 150-15; Clark, Reading, UK). Pipettes resistance was 1.3–1.7 MΩ. Each patch pipette was controlled by a micromanipulator (Narishige, Kyoto, Japan) and connected to the Axopatch 200B amplifier (Axon Instruments, Union City, CA). Voltage-clamp protocol generation and data acquisition were achieved by two outputs and inputs of the analog-to-digital/digital-to analog interfaces (Digidata 1200; Axon Instruments, Union City, CA) and pClamp 6 software (Axon Instruments, Union City, CA). Currents were low-pass filtered at 1 kHz with a Bessel filter; the sampling interval was 0.6 ms. The protocol of the stimulation and the recording procedure has already been reported in details in previous researches (Barrio et al., 1991; Valiunas et al., 2001; Formigli et al., 2005a; Gonzàles et al., 2007; Meacci et al., 2010). In brief, at the beginning of the experiment, the membrane potentials of cell 1 (V1) and cell 2 (V2) were clamped to the same value, V1 = V2. Then, V1 was modified to create the trans-junctional voltage (Vj), Vj = V2 − V1. From a holding potential (HP) of 0 mV, cell 1 was stepped using a bipolar pulse protocol starting at Vj = ±10 mV and ongoing at 20-mV increments up to ±150 mV. Test pulses lasted 5 s. Any current recorded from cell 1 was the result of two components: the membrane current of cell 1 and the transjunctional current (Ij). The currents recorded from cell 2 are indicated as −Ij. After the giga-seal was made, we electronically compensated (65–85%) the series resistance of electrodes 1 and 2, Rs1 and Rs2. The amplitude of Ij was determined at the beginning (instantaneous current, Ij,inst) and at the end of each pulse (steady state current, Ij,ss). These values were used to calculate the related conductance Gj,inst and Gj,ss. The Gj,ss voltage dependence, estimated by the analisys of the Gj,ss−Vj plot, was best fitted by the Boltzmann function:

Gj,ss= (Gmax− Gmin)/{1 + exp[A(V j− V0)]}+Gmin,

where Gmax represents the maximal Gj,ss conductance and Gmin the residual conductance at the end of the voltage steps, A is the constant representing the voltage sensitivity, Vo is the transjunctional voltage halfway between Gmax and Gmin. Gj,ss was normalized to Gj,inst and plotted against Vj. For mathematical and statistical analysis of data (expressed as means ± SEM) we used Clampfit 9 (Axon Instruments, Union City, CA). A two-sample t-test was used to compare single parameters between two independent experimental groups. ANOVA with repeated measures was used for multiple comparisons, followed by the Bonferroni’s *post hoc* test. p< 0.05 was considered statistically significant, unless otherwise specified. Experiments were made at 22°C.

**RT-PCR**

RNA isolation - Total RNA was isolated by extraction with TRIREAGENT (Sigma Aldrich, Italy), according with the manufacturer’s instructions (Frati et al., 2015). Concentration and purity of extracted total RNA were evaluated by spectrophotometric determination of the absorbance at 260 and 280 nm, and the integrity confirmed by agarose gel electrophoresis. Reverse transcription and PCR analysis – First, to avoid genomic DNA contamination the samples were treated with 2–10 U DNase I (Sigma Aldrich, Italy). One µg of total RNA from WB-F344 cells were reverse-transcribed to single stranded cDNA using the commercially available cDNA Synthesis Kit (SuperScript® III cells Direct cDNA Synthesis Kits, Life technologies) according to the manufacturer’s instructions. Samples were incubated at 25°C for 10 min, at 37°C for 120 min and then at 85°C for 5 min in a thermal cycler (Perkin Elmer). In order to amplify Cx isoforms subtypes and GAPDH housekeeping gene, we choosed specific forward and reverse primers as following: GAPDH forward 5’-GGCAAATTCAACGGCACAGTC-3’ reverse 5’-TCGCTCCTGGAAGATGGTG-3’; Cx43 forward 5’- AACAGTCTGCCTTTCGCTGT-3’ reverse 5’-TCTGCTTCAGGTGCATCTCC-3’; Cx32 forward 5’- AAAATGCTACGGCTTGAGGG3’ reverse 5’- TGAAGACGGTTTTCTCG GTG -3’and Cx26 forward 5’-

ACTCCACCAGCATTGG AAAG-3’ reverse 5’-TGAGAGAT GGGGAAGTGGTG -3’; Real-time PCR - Quantitative real-time PCR was carried out using 7500 Fast Real-Time PCR System (Applied Biosystems) and Power SYBR Green PCR Master Mix (Life technologies), consisting in a specific set of primers and a fluorogenic internal probe. The expression of Cx genes were quantified in comparison with the housekeeping gene GAPDH. PCR amplifications were performed on cDNA samples corresponding to a final RNA concentration of 100 ng. PCR was performed in a total volume of 20 µl containing 2× PCR Master mix (Life technologies). Reaction conditions were as follows: 95°C for 10 min, followed by 40 cycles at 95°C for 30 s, 60°C for 30 s, 72°C for 45 s (fluorescence was collected during the elongation step), finally followed by 95°C for 15 s, 60°C for 60 s, 95°C for 15 s, and 60°C for 15 s for the dissociation analysis. PCR amplifications were run in duplicates. Blank controls, consisting in no template (water) or RT negative reactions, were performed in each run. The results of the real-time PCR were presented as Ct values, where Ct was defined as the PCR threshold cycle at which amplified product was first detected. All values were normalized to the GAPDH housekeeping gene expression.

**Western blotting**

Following the indicated treatments, WB-F344 cells were collected in Lysis buffer as reported in (Pierucci et al., 2017; Meacci et al., 2010). The protein samples were subjected to SDS-PAGE (sodium dodecyl sulfate-polyacrylamide gel electrophoresis) before being transferred onto a nitrocellulose membrane (Hybond ECL membranes, GE Healthcare; Little Chalfont, UK) and immunodetected with specific antibodies: the MAB3067 anti-Cx43 antibody (Millipore; Billerica, MA, USA), polyclonal anti-Cx43 (Sigma Aldrich, Italy), and mouse monoclonal anti-Cx32 antibody (Santa Cruz) and mouse monoclonal anti-Cx-26 (Sigma Aldrich, Italy). Subsequently, an anti-β-actin from Santa Cruz was applied. For chemiluminescence detection, either the ECL Plus reagent (GE Healthcare) or Lumina from Millipore was used (Millipore; Billerica, MA, USA).

**PP2A enzymatic activity determination.**

PP2A enzymatic activity was determined by PP2Ac immunoprecipitation and phosphatase assay based on malachite green reaction accordingly to Immunoprecipitation Phosphatase Assay Kit protocol (Upstate) with minor modifications. Briefly, 70-100 µg of total cellular protein was incubated with either protein A agarose slurry alone or in the presence of anti-PP2Ac Ab (clone 1D6; Upstate) at 4°C for 2 hours. Agarose-bound immune complexes were collected and washed with TBS and once with optimized Ser/Thr buffer. The immuno complex was then resuspended in 20 µl Ser/Thr buffer and incubated with the substrate for PP2Ac, the phosphor-peptide K-R-pT-I-RR, at 30°C for 20 min. Supernatants (30 µl) were transferred in 96-well plate, and the released phosphate was quantified by adding 100 µl malachite green phosphate to the p96 well and reading the plate at 650 nm after 15 min. The absorbance of the reactions was corrected by subtracting the blank (absorbance of sample treated with anti-IgG Ab). Phosphate concentrations were calculated from a standard curve created using serial dilutions of a standard phosphate solution as described in the kit protocol.

**Cell transfection with siRNA**

For transfection of WB-F344 cells, we used the previously established protocol (Pierucci et al., 2017; Frati et al., 2017; Meacci et al., 2008). Briefly, cells were plated in DMEM without antibiotics and transfections were performed using siRNA duplexes directed against PP2A mRNA sequence (CACCAUACUCCGAGGGAAU[dT] [dT]) (Sigma Aldrich, Italy) or control siRNA-(SCR, scrambled) from Santa Cruz Biotechnology. The transfections were carried using siRNA (50nM) and Lipofectamine 2000 (Invitrogen, Life Technologies Italia Fil. Life Technologies Europe BV, Monza, Italy), according to the manufacturer’s instructions. Transfection mix was removed 6 h later, and WB-F344 cells were cultured for further 24-48 h in DMEM containing fetal calf serum before the indicated treatments.

**Ceramide Kinase Activity determination**

The ceramide kinase activity (CerK) was assayed as described previously (Mitsutake and Igarashi, 2005) with some modifications. Briefly, WB-F344 cells treated with vehicle (0.05% DMSO) or PCB153 (20 mM) were lysed in a buffer containing 10 mM HEPES, 1 mM dithiothreitol, 40 mM KCl, and protease inhibitor mixture (Sigma Aldrich, Italy). Each lysate was incubated for 30 min at 30 °C in a reaction mixture containing 20 mM HEPES, 80 mM KCl, 1 mM cardiolipin, 1.5% -octylglucoside, 0.2 mM diethylenetriaminepentaacetic acid, 20 µM [32P]ATP, and 40 µM Cer (C18:0, d18:1). Lipids were extracted and separated on Silica Gel 60 high performance TLC (HPTLC) plates (Merck, Darmstadt, Germany) using chloroform/acetone/methanol/acetic acid/water (10:4:3:2:1, v/v) as the solvent system. Bands corresponding to C1P were quantified using an imaging analyzer (Beckman).

**Measurements of cellular [3H]-sphingolipids**

Cell monolayers were incubated with 40 µCi/ml of [3H]palmitate complexed with 1 mg/mL fatty acid-free bovine serum albumin for 24 h in serum free-medium at 37° C. After treatment with either PCB153 or the vehicle (0.05% DMSO) for the indicated times, Cer and C1P were extracted separated by thin layer chromatography (TLC) (Merck, Darmstadt, Germany) using chloroform/methanol/acetic acid (9:1:1, v/v/v) solution and dried. They were then developed for their full length with petroleum ether/diethylether/acetic acid (60:40:1, v/v/v) as described in Bini et al. (2012), Frati et al., 2015, Pierucci et al., 2017) The position of Cer was identified after staining with I2 vapour by comparison with authentic standards. Radioactivity of the samples, obtained by scraping the Cer and C1P spots from the plates, was quantified by liquid scintillation counting.

**Statistical analysis**

In immunoblot experiments, densitometric analysis of the bands was performed by ImageJ software (N.I.H.) (http://rsb.info.nih.gov/ij/index.html). Band intensity was reported as relative percentage (means ± S.E.M.), obtained by calculating the ratio of specific protein on β−actin intensity and normalizing to control, set as 100. Mathematical analysis of electrophysiological data was performed by Pclamp 9 software (Axon Instruments, CA, USA). Statistical significance was determined by Student’s t test or one-way ANOVA and Bonferroni’s post-test. A value of p<0.05 was considered significant. Results are given as means ± S.E.M.
