# Supplementary material for: Ceramide/protein phosphatase 2A axis is engaged in gap junction impairment elicited by PCB153 in liver stem-like progenitor cells
Source: Mol Cell Biochem. 2021 Apr 10;476(8):3111–26. doi: 10.1007/s11010-021-04135-z (PMC8263450; doi:10.1007/s11010-021-04135-z)
Supplement: Supplementary file 4 — Supplementary file4 (PPTX 80 KB) [file 11010_2021_4135_MOESM4_ESM.pptx]

## Slide 1
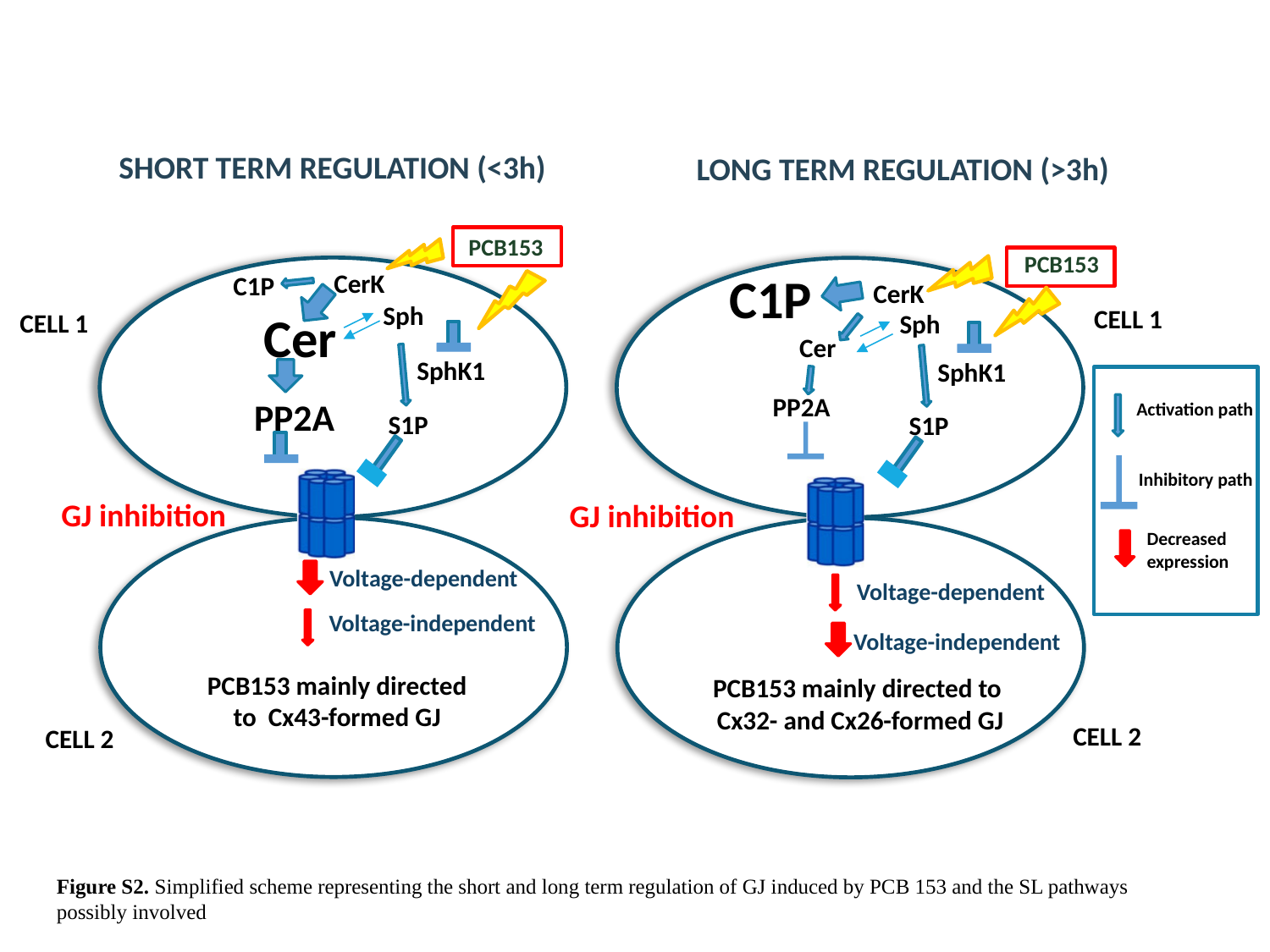

SHORT TERM REGULATION (<3h)
LONG TERM REGULATION (>3h)
PCB153
 CerK
PCB153
GJ
 C1P
CerK
C1P
Sph
CELL 1
Cer
CELL 1
Sph
Cer
SphK1
SphK1
 PP2A
PP2A
Activation path
 S1P
 S1P
 Inhibitory path
GJ inhibition
GJ inhibition
Decreased expression
Voltage-dependent
Voltage-dependent
Voltage-independent
Voltage-independent
PCB153 mainly directed to Cx43-formed GJ
PCB153 mainly directed to Cx32- and Cx26-formed GJ
CELL 2
CELL 2
Figure S2. Simplified scheme representing the short and long term regulation of GJ induced by PCB 153 and the SL pathways possibly involved
